# Distraction by a monotube fixator to achieve limb lengthening: predictive factors for tibia trauma

**DOI:** 10.1186/1752-2897-7-3

**Published:** 2013-05-14

**Authors:** Olayinka O Adegbehingbe, Owolabi D Ojo, Paul O Abiola, Abimbola L Ariyibi, Lawrence M Oginni, John A Obateru

**Affiliations:** 1 Department of Orthopedic Surgery & Traumatology, College of Health Sciences, Obafemi Awolowo University, Ile-Ife, Nigeria; 2 Federal Medical Centres, Department of Orthopedic Surgery, Ido-Ekiti, Nigeria

**Keywords:** Bone gap, Bone transport, Limb lengthening, Trauma, Rural Orthopedics Practice

## Abstract

**Background:**

Management of post trauma tibia bone gap varied with orthopedic surgeons’ experience and tools available. Study aims to determine predictive factors for distraction by a monotube fixator (DMF) outcome in post tibia trauma limb length discrepancy.

**Methods:**

A prospective descriptive cross sectional study of post traumatized tibia bone gap and limb length discrepancy patients at tertiary hospitals. Patient’s informed consent and institutional ethical committee approval were obtained. Bio-data, clinical and healing indexes were documented. DMF was applied for patient that met inclusion criteria. The Statistic tests used included the Chi-square, the Student’s two-tailed *t* test, and the Wilcox on rank-sum test when appropriate. Mantel-Haenszel Common Odds Ratio (OR) and 95% confidence intervals for poor outcome potential risk factors were recorded. Bivariate correlation and logistic regression were evaluated. Significance level was set at a p value <0.05.

**Results:**

Thirty-six patients with mean age, 37.2 ± 10.3 year and male/female ratio of 1:1.25 had DMF applied. Motorcycle accident accounted for 50.0% of patients and diaphyseal segment was most commonly affected 25 (69.4%). The mean bone lengthened was 10.1 ± 4.0 cm (range: 5-21 cm) and mean duration of bone transport was 105.6 ± 38.2 days. The means of rate of distraction, healing index and percentage of lengthening were 0.99 ± 0.14 mm/day, 15.6 ± 4.3 days/cm and 38.0 ± 14.3 respectively. The mean follow up was 9.7 ±4.9 months (range: 2–17.0). Per operative complications varied and outcome was satisfactory in 30 (83.3%). Obesity (p <0.0001), multiple surgery (p = 0.012) and transfusion (p = 0.001) correlated to poor outcome. Percentage lengthening ≥ 50%, bone gap >10 cm, anemia, blood transfusion, general anesthesia administration, distraction rate >1 mm/day, osteomyelitis and prolong partial weight bearing were significant predictive factors for poor outcome in post traumatic tibia distraction.

**Conclusion:**

Distraction by a monotube fixator appears effective in achieving correction >38.0% original tibia lengthening following traumatic bone gap. Predictive factors for poor outcome were useful for prognostication.

## Background

The management of long bone fractures with significant bone gap and limb length discrepancy can pose a major challenge to even the most experienced trauma surgeon. In the United State, the Wagner methods of limb lengthening were utilized predominantly from 1970 to 1990; however, it was associated with a high rate of complications including infection, non-union, and failure of fixation [1-3]. In the early 1980s, Soviet Union and European surgeons began to lengthen bones with the use of slow, gradual distraction after an osteotomy or corticotomy, obviating the need for bone grafting and decreasing the prevalence of delayed consolidation and non- union [4].

The major limiting factor for bone distraction and lengthening is the resistance offered by the soft tissues [5]. The difference in the adaptability of the newly formed bone and established soft tissues during distraction osteogenesis generate resistive tension, termed distraction-resisting forces [6]. To solve this problem of soft tissue resistance, Abbot, in 1939 introduced excessive dissection of the muscles and fasciae [7]. Ilizarov, in 1956 modified his apparatus according to the differences in muscular resistance and balance at various limb segment levels [8]. Currently, soft tissue is the main factor in limb lengthening causing complications such as muscle contractures, joint stiffness, joint subluxations, and axial deviations [9]. Because of the challenges of the soft tissue associated distraction-resisting forces, modifications and innovations have been made in limb lengthening techniques [10,11].

An accurate and precise non-invasive method of quantifying new bone formation is essential for the assessment of bone healing during limb lengthening. Plain film documentation is usually possible to identify the new bone as linear streaks appearing between three and nine weeks after distraction is initiated [12]. Nevertheless, the surgeon must often continue distraction for several weeks without visual documentation of new bone formation.

Distraction by a monotube fixator to achieve limb lengthening was popularized recently in Nigeria and predictive factors for poor outcome in tibia bone gap from trauma have not been evaluated. We assumed the hypothesis there was no difference in outcome of tibia bone gap sequel to trauma managed using the same model of monotube fixator. Study justification was based on the varied reports on distraction osteogenesis in terms of quality, criteria used for evaluation, size of the study group, characteristics of the patients, techniques of lengthening and type of fixation used. The variability of the designs makes it difficult to compare the different studies or to draw conclusions on predictive factors for poor outcome [4].

## Methods

A prospective descriptive cross sectional study of patients with post tibia trauma and limb length discrepancy presenting at Obafemi Awolowo University Teaching Hospitals Complex, Ile Ife and Federal Medical Centre, Ido-Ekiti, Southwestern Nigeria. Patient’s informed consent and institutional ethical committee approval were obtained.

The main inclusion criterion was unilateral limb- length discrepancy from traumatic tibia bone gap (≥5 cm). Adequate wound debridement where necessary in open fracture was done. Excluded were patients with simultaneous congenital deformity correction and lengthening, patients diagnosed as having generalized ligamentous laxity and patients who have been managed with bifocal tibia or proximal fibular osteotomies in the same limb.

The demographic data, clinical and radiological information were documented for each patient.

The age at the time of lengthening, indication for limb lengthening, number of days from the time of osteotomy to the onset of distraction, number of days for distraction osteogenesis, time needed for the callus maturation, total number of days for which the monotube fixation was used before removal, and total duration of treatment which included the duration for the fixator was used plus the duration of subsequent immobilization. Obesity was defined as patients having Body Mass Index ≥ 30 kg/m^2^.

Plain X-Ray radiograph for all patients were of the same machine, technique and distance. The baseline length of tibia bone was measured on the preoperative radiograph. The immediate postoperative X-ray film was used to document the level of osteotomy as proximal or distal metaphyseal or diaphyseal of the Tibia. The length gained was measured on the radiograph taken after the completion of the distraction. Radiographs were evaluated after the discontinuity of the monotube fixation for non union, fracture or deformity of the lengthened bone.

The distraction gap was measured precisely on plain film through centralization of the radiographic beam unto to the distraction site and magnification is maintained for all the patients. A radiopaque ruler was fixed by Velcro bands to the limb for radiograph. Early indicator of an incomplete corticotomy or premature consolidation was failure of the osetotomized gap to separate after two weeks of distraction. To improve precision a line was drawn on radiographic request forms to illustrate the exact corticotomy level in relation to the linear ray clamps. The extent of distraction gap achieved was calculated at each clinic visit by the surgeon as a way of monitoring patient’s compliance with specific instructions moderating the distraction rate.

The rate of distraction in millimeters per day was determined by dividing the length gained by the total number of days of distraction. The percentage of lengthening was calculated by dividing the length gained by the total length of bone as measured on the radiographs. The healing index [2] was calculated by dividing the total duration of treatment (the duration for which the monotube fixator was utilized plus the duration of any subsequent immobilization) in days by the total amount of length gained in centimeters or by the percentage of length gained.

### Distraction by a monotube fixator and lengthening protocol

The monotube fixator components from Bombay, India were used for all the patients because it was affordable, accessible and require simple skill to apply for limb lengthening.

All patients had one lengthening osteotomy performed in each tibia. The lengthening protocol closely followed that described in previous studies [2] under appropriate anesthesia, general or spinal. Patients were encouraged to walk, with partial weight bearing, as often as possible. After waiting period of 14 days, distraction was begun at the rate of one millimeter per day in four 0.25 millimeter increments. The pin sites were cleaned daily with povidone iodine. When pin site infection was established, treatment with oral antibiotics administration was begun based on culture sensitivity.

Patients were instructed to keep the knee in full extension as much as possible during the day. A solid ankle foot orthotics was applied at night to prevent tightness of the heel cord. After achieving the desired limb length, the monotube fixator was locked. In few occasions when the maximum length of the monotube fixator was reached before the calculated desired limb length was attained, the original fixator bar was exchanged for a longer one.

Physical therapy with active-assisted and passive range of motion exercises was started. The monotube fixator and the pins were removed under anesthesia to prevent deformation of the callus. All patients required additional immobilization in an above-the- knee cast for an average of 90 days and axillaries crutches. The follow up was for at least midterm period after monotube fixator removal to detect any post operative complication.

The results of the clinical examination at the time of monotube fixator removal were recorded, and all patients were examined at the time of the latest follow-up by at least two of the authors (OOA, OOD, and POA) with similar professional experience. In this study unsatisfactory/poor outcome refer to presence of either percentage lengthening that is not equal to the normal tibia length, severe bone infection and or non union after distraction osteogenesis.

### Statistical analysis

Statistical analysis was performed using Statistical package SPSS version 16. The Statistic tests used included the Chi-square, the Student’s two-tailed *t* test, and the Wilcox on rank-sum test when appropriate. Mantel-Haenszel Common Odds Ratio (OR) and 95% confidence intervals for poor outcome potential risk factors were recorded. Logistic regression of linear correlation model with outcome as dependent variable and independent variables as predictors was evaluated. Significance level was set at a p value <0.05.

## Results

Between September 9, 2008 and August 29, 2011 thirty-six traumatized patients had significant tibia bone gap and limb length shortening corrected with distraction by a monotube fixator. Sixteen males (44.4%) and twenty females (55.6%) with male/female ratio of 1:1.25 met the inclusion criteria. The mean age at presentation was 37.2 ± 10.3 year (range: 21-54 year) and 24 (66.7%) were between age 17–44 year. The majority, 21 (58.3%) were unskilled and fifteen were skilled workers (41.7%). The occupation pattern revealed public service, 10 (27.8%), farming/trading, 9 (25%), schooling, 8 (22.2%), police force, 5 (13.9%) and commercial motorcycling, 4 (11.1%). Road traffic accident (RTA) was the most common cause of tibia bone gap, given 30 out of 36 patients and motorcycle injury represented 50.0% of all traffic associated tibia traumas. The Table 1 shows the characteristic features of trauma patients with significant tibia bone gap.

**Table 1:** Characteristics of trauma patients with tibia bone gap

**Features**	**Frequency (%)**	**Significant level (p value)**
**Age (year)**	37.2 ±10.3	0.404
**Sex**		
**Male**	16.0 (44.4)	0.134
**Female**	20.0 (55.6)	
**Occupation**		
**Skilled**	15.0 (41.7)	0.650
**Unskilled**	21.0 (58.3)	
**Etiology**		
**RTA- MCA**	18.0 (50.0)	0.007
**MVA**	8.0 (22.2)	
**Pedestrian**	4.0 (11.1)	
**Occupational hazard**	6.0 (16.7)	
**Body Side Affected**		
**Right**	12.0 (33.3)	0.030
**Left**	24.0 (66.7)	
**Hospital Stay**	109 ± 59.9	
**< 28 day**	2.0 (5.6)	0.716
**≥ 29 day**	34.0 (94.4)	
**Follow up period**	9.7 ± 5.3	0.021
**0-6 months**	14.0 (38.9)	
**7-12 months**	10.0 (27.8)	
**13-18 months**	12.0 (33.3)	

N.B: RTA = Road Traffic accident, MVA = Motor Vehicle Accident, MCA = Motor Cycle Accident.

Plain radiograph of affected limbs shows segmental comminuted fractures of varied degrees of tibia bone gap in 26 (72.2%) patients and 27.8% had comminuted fracture with bone gap. The involved proximal third or middle third segments had more than 50% of cortical circumference loss at presentation. The tibia diaphyseal was mostly affected in 25 (69.4%) patients and 27.8% had their metaphyseal-diaphyseal segment involved.

The most preponderant body side affected was the left tibia 24 (66.7%) and the right tibia in twelve cases. Six patients had poor outcome after tibia distraction osteogenesis and limb lengthening, four out of six involved the right tibia (OR = 11.5, 95%CI = 1.11-118.77, p < 0.017). On presentation to the hospital majority of the patients, 72.2% had normal haemogram ≥10 gm/dl and ten patients were admitted with haemogram below 10 gm/dl. Fourteen patients (38.9%) were transfused with homologous blood and 27.8% patients required multiple blood transfusion (p < 0.001). Preoperative anemia (OR = 5.20, 95%CI = 1.12-24.08, p < 0.020) and blood transfusion (OR = 7.8, 95%CI = 1.0-60.0, p < 0.014) were predictive of patients’ poor outcome. Table 2 shows per operative features of trauma patients with tibia bone gaps on distraction by a monotube fixator to achieve limb lengthening.

**Table 2:** Per operative features of patients with tibia bone gaps distracted by a monotube fixator

**Features**	**Frequency (%)**	**Chi-square *X***^**2**^	**Significant level (p value)**
**Preoperative Packed Cell Volume (%)**	34.1 ± 6.5	27.00	0.001
**< 30.0**	10.0 (27.8)		
**≥ 30.0**	26.0 (72.2)		
**Tibia segment**		7.20	0.027
**Diaphyseal**	25.0 (69.4)		
**Metaphyseal/diaphyseal**	11.0 (31.6)		
**Number of Operation performed**	1.7 ± 0.8	8.80	0.012
**One**	18.0 (50.0)		
**Two**	8.0 (22.2)		
**Three**	10.0 (27.8)		
**Bone gap (cm)**	10.1 ± 4.1	4.41	0.049
**5.0-9.9**	20.0 (55.6)		
**10.0-14.9**	14.0 (38.9)		
**20-24.9**	2.0 (5.6)		
**Anesthesia type**		23.04	0.000
**Spinal**	30.0 (83.3)		
**General Anesthesia**	6.0 (16.7)		
**Intra Operative Blood Loss (ml)**	403.9 ± 150.7	18.9	0.042
**≤ 499.0**	26.0 (72.2)		
**≥ 500.0**	10.0 (27.8)		
**Number of blood pints transfused**	2.0 ± 0.7	14.00	0.001
**One**	4.0 (11.1)		
**Two**	6.0 (16.7)		
**Three**	4.0 (11.1)		
**Co-morbidity**		0.046	0.829
**Head injury**	2.0 (5.6)		
**Diabetic mellitus**	2.0 (5.6)		
**Obesity**	4.0 (11.1)		

Spinal anesthesia was administered in 30 (83.3%) and six patients (16.7%) had general anesthesia before tibia corticotomy and monotube fixator application. Five of the patients that received general anesthesia had poor outcome as compared to spinal anaesthetized patients (OR = 25, 95%CI = 3.52-177.47, p < 0.0001). Partial fibulectomy was routinely performed for all patients. Partial thickness skin grafting was indicated in thirty patients and four had poor outcome (13.3%, *X*^2^ = 1.44, p > 0.230).

**Figure 1:**
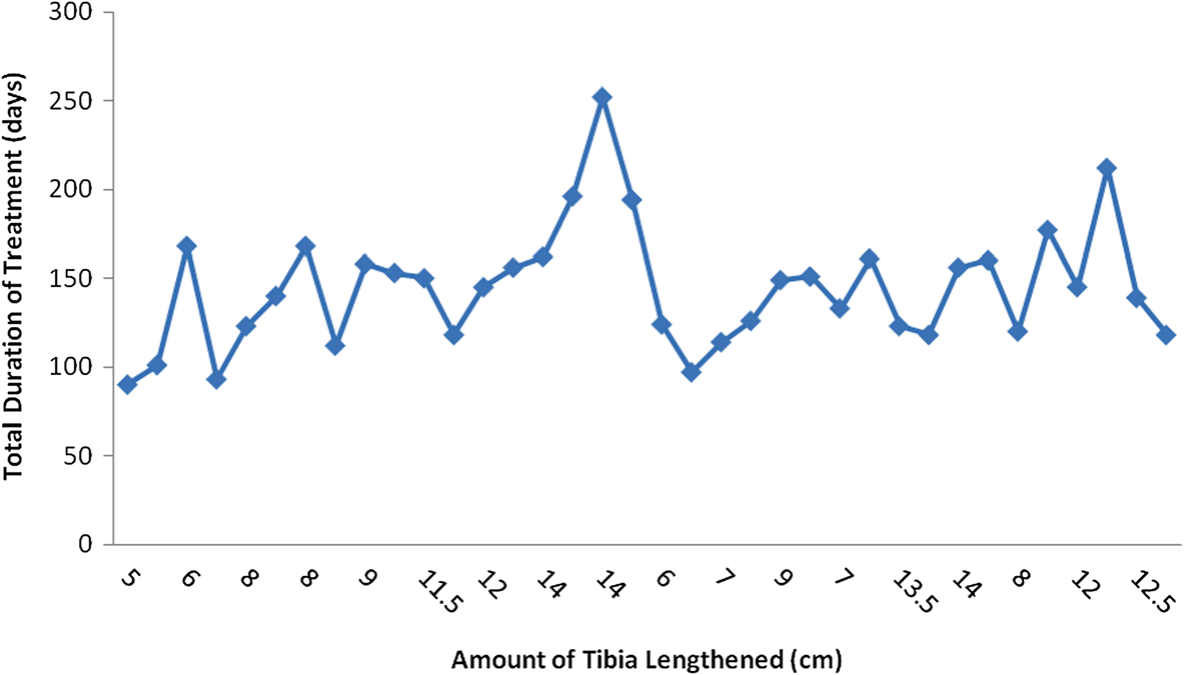
Total duration of treatment (days) versus amount of tibia lengthened (cm).

Limb lengthening outcome was not affected by age, sex, and occupation (p > 0.050). Young patients’ age group, 20–24 year had lower post operative complications compared to older age group (p = 0.001). However, no age group difference exist in pattern of post operative complications (*X*^2^ = 2.250, p = 0.134). The percentage lengthening were <25%, 25-50% and >50% which occurred in 22.2%, 50.0%, and 27.8% of patients respectively (*X*^2^ = 28.8, p = 0.036). Table 3 shows bivariate correlation of healing indexes in tibia distraction by monotube fixator and limb lengthening outcome. A tibia bone gap > 10 cm (OR = 8.6, 95%CI: 0.89-83.7, p = 0.036), with rate of tibia distraction >1 cm/day (OR = 0.13, p = 0.014) and percentage lengthening ≥50% (OR = 8.0, 95CI: 1.17-54.49, p = 0.020) were predictive of poor outcome. Figure 1 shows total duration of treatment as related to length of tibia lengthened (cm).

**Table 3:** Healing indices of tibia distraction osteogenesis and limb lengthening outcome

**Features**	**Mean ± SD (range)**	**Significant level (p value)**
**Length of Bone Gap (cm)**	10.1 ± 4.0	0.092
**Duration of Bone transport (day)**	105.6 ± 38.7 (range: 41–170)	0.633
**Percentage Lengthening**	38.0 ± 14.3 (range: 19.0-70.0)	0.014
**Healing index (days/cm)**	15.6 ±4.3 (range: 9.2-28.0)	0.791
**Healing index (days/% lengthening)**	4.2 ±1.2 (range: 2.7-8.1)	0.039
**Rate of distraction (mm/day)**	0.99 ± 0.14 (range: 0.65-1.27)	0.014
**Total duration of treatment (day)**	148.8 ± 39.9 (range: 90.0-252)	0.315
**Hospital stay (days)**	109.0 ± 59.9 (range:22–252)	0.836

**Figure 2:**
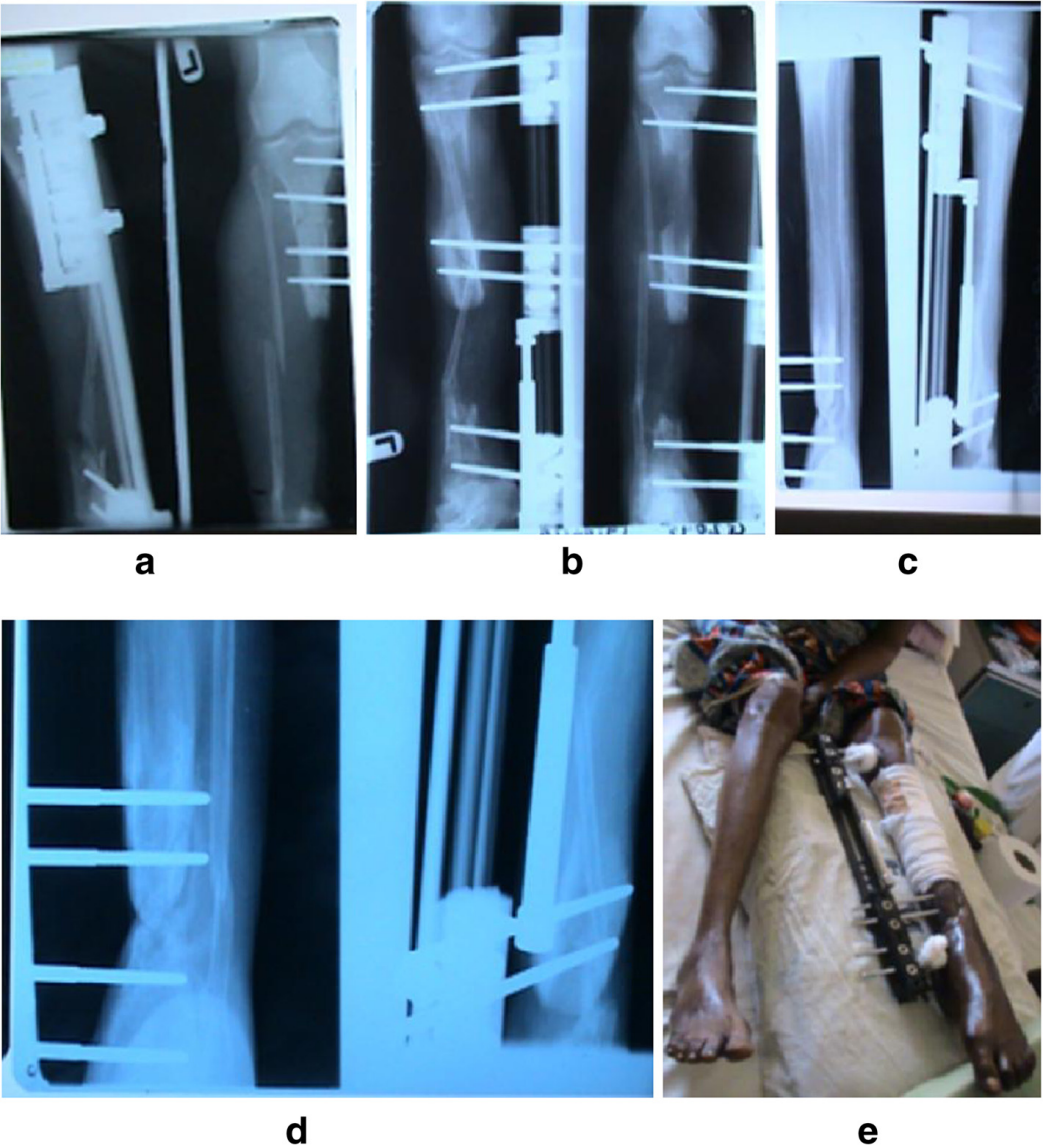
**A monotube fixator applied on patient's left tibia with 21 cm bone gap achieving 70% limb lengthening. a**. Tibia bone gap **b**. post corticotomy **c**. post distraction **d**. Docking **e**. photo of the patient with DMF.

In this study, no patient had knee ligament laxity at full extension, and no patient had ankle joint or distal tibia-fibula joint laxity. No patient reported pain or tenderness at the fibular head with direct palpation or rotation of the foot. No patient reported a subjective sense of instability at the knee joint during walking or running. No peroneal nerve abnormality was found, with all patients recovering full range of motion and power at the knee and ankle joints at the end of the physiotherapy care. Intra-operative iatrogenic fracture of the tibia occurred in four patients which were recognized during surgery and managed conservatively. After removal of the monotube fixator, seven patients had deformations of the callus without a fracture. An above knee scotch cast was applied for each of the iatrogenic fractures and callus deformation. No surgical soft tissue release was performed for the treatment of knee stiffness because stiffness consistently responded well to physiotherapy. Figure 2a-e depicts the radiological progression of the patient with 21 cm representing 70% tibia lengthening.

Among co-morbidity factors, 3 out of 4 obesities (p < 0.0001) correlated with unsatisfactory outcome. The commonest post operative complication was pin tract infection followed by disuse osteoporosis as shown in Table 4. Nineteen of the thirty-two pin tract infections necessitated intravenous administration of antibiotics and removal of thirteen pins. Osteomyelitis (OR = 12.4, 95%CI = 3.02-50.80, p < 0.000) and prolong duration of partial weight bearing ≥6 months (OR = 5.8, 95%CI = 4.23-795.24, p < 0.000) were significant predictive factors for poor outcome as shown in Table 5. Secondary surgeries were indicated in exchange of eleven monotube fixators, removal of pins following infection, nine manipulations to correct angulations, open osteoclasis for premature consolidation and three elongation of Achilles tendon to treat equines deformity. Thirty out of thirty-six patients’ outcome was satisfactory representing 83.3% and it was poor in six patients (16.7%). Residual limb length discrepancy occurred in six patients and three were correctable with raised shoes. One patient travel outside the country for further care and two patients await further lengthening procedure to achieve equality of the limb.

**Table 4:** Complications of tibia distracted by a monotube fixator in post trauma patients

**Complications**		**Frequency (%)**	**Chi-Square *X***^**2**^	**Significant level (p value)**
Intra operative	Iatrogenic fracture	4.0 (11.1)	0.090	0.343
Post operative	Pin tract infection	32.0 (88.9)	26.10	0.002
	Osteomyelitis	5.0 (13.9)		
	Equines	19.0 (52.8)		
	Out toeing	2.0 (5.6)		
	Knee stiffness	12.0 (33.3)		
	Malunion	10.0 (27.8)		
	Premature consolidation	2.0 (5.6)		
	Disuse atrophy	30.0 (83.7)		
	Sudeck atrophy	1.0 (2.8)		

**Table 5:** Predictive factors for tibia limb lengthening poor outcome distracted by a monotube fixator

**Variables**	**Odds ratio**	**95% confidence interval (CI)**		**Significant level-p value**
		**Lower bound**	**Upper bound**	
**Right Tibia Affectation**	11.5	1.114	118.707	0.017
**Haemogram (< 10 gm/dl)**	5.2	1.123	24.080	0.005
**Require Blood Transfusion**	7.8	1.022	60.414	0.014
**General Anesthesia**	25.0	3.522	177.470	0.0001
**Bone gap (> 10 cm)**	8.6	0.891	83.770	0.036
**% Lengthening (≥ 50%)**	8.0	1.174	54.490	0.020
**Rate of distraction (> 1 mm/day)**	11.6	1.188	114.590	0.014
**Partial weight bearing (> 6 months)**	58.0	4.23	795.246	0.0001
**Osteomyelitis**	12.4	3.027	50.804	0.0001

## Discussion

In this study, the main indication for distraction by a monotube fixator to achieve limb lengthening in Southwestern Nigeria was RTA. At least fifty percent of the patients having significant tibia bone gap were victims of motorcycle crashes. Post tibia trauma limb length discrepancy reported here was in sharp contrast to earlier reports from Europe, Canada, China and South America, where achondroplasia, idiopathic short statures and other skeletal dysplasia were the main limb lengthening indicators [13,14]. In 2004, there were 14,279 reported road crashes and 16,897 people were injured in Nigeria. An increasingly large proportion of these burdens were due to motorcycle crashes many of which were used for commercial commuting (Federal Road Safety Commission, 2006 [15]. This study from Table 1 has shown that motorcycle crash related lower limb injuries are still common in Nigeria. This was in keeping with the rising trend of road traffic injuries all over the world but far more in low to middle-income countries [16]. The reason for majority of the tibia bone gaps might be related to the unprotected and exposed lower limb on the motorcycle. Also, it could be explained from the established risky behaviors among drivers and motorcycle riders involved in road crashes in southwest Nigeria. These were compounded by poor road conditions and chaotic traffics [17].

The demographic characteristics of our study population were similar to that reported by Oluwadiya et al. [17]. Majority of the patients sustained their injuries on their way to work or school probably reflecting impatience and or over speeding with severe limb injuries significant enough for limb lengthening. Accidents related to road traffic, industrial machinery or farming can result in mangling injuries with bone exposure and bone gap [18]. Almost all studies in the literature have supported the view that sex has no effect on distraction osteogenesis [19] similar to our results.

In this study, the tibiae were lengthened an average of 10 cm, or ≥38 percent of the original tibia length. This was higher than the maximum 6 cm; representing 21% of the original tibia length earlier reported [4]. The amount of tibia bone loss in RTA was unrestricted by skeletal maturity as shown in Figure 2a-e, but reflected the level of severity of injury and or amount in tibia bone gap that patient in our environment would give consent for distraction by monotube fixation.

Tibia lengthening in patients who were older than twenty-five years were associated with higher rates of post operative complications as compared to younger ages. The more stretchable ligaments, better adaptability of young muscles and soft tissues could be a factor for young patients to have fewer complications during limb lengthening [9].

The significant predictive factors in Table 5 could explain partly clinical pattern in Figure 1. The unsatisfactory outcome in post trauma tibia distraction and limb lengthening include affectation of right body side, preoperative anemia haemogram, general anesthesia administration and blood transfusion. The right leg is usually the flight limb used by many people to jump out from danger or escape from crash notably traffic accidents. When the right leg is trapped or crushed in an accident could suggest a more severe injury and bone defect that could signal poor outcome. A preoperative anemia might be reflecting pre morbid nutrition or severe damage to blood vessels that could predict the bone defective healing outcome. In general, except when contraindicated the lower limb surgeries routinely are usually performed under spinal anesthesia including monotube fixator application. However, when general anesthesia as shown in Table 2 was indicated primarily for limb lengthening procedure could predict poor outcome. Because, co- morbidity conditions like obesity, diabetic mellitus and anemia are often present.

The reported rates of complications for tibia lengthening with the use of distraction osteogenesis have been as high as 167 percent [20]. It appears our study complications shown in Table 4 are concordant with other reported rates. Direct comparison of our results with previously published studies was impossible because of differences in the inclusion/exclusion criteria such as classification of complications, the diagnoses, the presence of associated limb deformities, and the amount that the segments were lengthened. Our results revealed osteomyelitis could predict poor outcome probably from further bone loss. Also, among obese patient, distraction-resting force from soft tissue tension is high and could limit the distraction gap and slow the distraction outcome.

The tibia healing indices in Table 3 were related [i] to the amount of length gained in centimeters depicted in Figure 1 and [ii] to the percentage of length gained; a relative increases in length can be expected to decrease the healing indices [5,13,21]. Our study outcome of tibia distraction osteogenesis was satisfactory when the percentage lengthening was below 50% and poor outcome predicted when > 50% of original tibia length. Because the early phase of tibia distraction generate tension on the ligaments of the proximal tibia-fibula joint, causing proximal tibia-fibula joint distraction. Later, when the tension in these ligaments is high, tibia valgus angulations’ starts to increase rapidly. Thus, the distraction-resisting forces can be said initially to pull on the tibia-fibula joints and then later to exert a deforming force on the tibia [21] associated knee laxities and poor outcome. As documented here, duration of partial weight bearing could be early indicator to poorer outcome in clinical scenario after distraction by a monotube fixator.

Limitations of study include lack of validated outcome instruments to compare present results and absence of external validity of the protocol together with smallness of subjects. However study was multicentre and strict inter observer agreement on outcome measures applied could reduce the effects of aforementioned limitations on the study inferences.

## Conclusion

The prognostication of outcome following the use of distraction by a monotube fixator to achieve limb lengthening in post tibia trauma with significant bone gaps is a major concern for trauma specialist practitioners.

This study has added to knowledge, predictive factors for poor outcome in post traumatic tibia distraction by a monotube fixator. DMF appeared to achieve correction ≥ 38.0% tibia bone gap. Percentage lengthening ≥ 50%, bone gap >10 cm, anemia, blood transfusion, general anesthesia administration, distraction rate >1 mm/day, osteomyelitis and prolong partial weight bearing were significant predictive factors for poor outcome useful for prognostication.

## Abbreviations

RTA: Road traffic accident; MCA: Motor cycle accident; MVA: Motor vehicle accident.

## Competing interests

The authors declare that they have no competing interests.

## Authors’ contributions

AOO -conceived the study, participated in the design, acquisition of data, analysis and interpretation of data, have been involved in drafting the manuscript,critically revising it for important intellectual content and have given final approval of the version to be published. OOD-participated in the design, acquisition of data, have been involved in drafting the manuscript,critically revising it for important intellectual content and have given final approval of the version to be published. APO: participated in the design, acquisition of data, have been involved in drafting the manuscript,critically revising it for important intellectual content and have given final approval of the version to be published. AAL: participated in the design, acquisition of data, have been involved in drafting the manuscript,critically revising it for important intellectual content and have given final approval of the version to be published. OLM: participated in the design, acquisition of data, interpretation of data, have been involved in drafting the manuscript,critically revising it for important intellectual content and have given final approval of the version to be published. OJB: participated in the design, acquisition of data, analysis and have been involved in drafting the manuscript, critically revising it for important intellectual content and have given final approval of the version to be published. All authors read and approved the final manuscript.

## Authors’ information

AOO: Senior Lecturer & Consultant Orthopedic Surgeon, Obafemi Awolowo University Teaching Hospitals Complex, Ile Ife Nigeria.

OOD: Head of Department & Consultant Orthopedic Surgeon, Federal Medical Centre, Ido Ekiti, Nigeria.

APO: Consultant Orthopedic Surgeon, Federal Medical Centre, Ido Ekiti, Nigeria.

AAL: Head, Department of Accident Emergency & Consultant Orthopedic Surgeon, Federal Medical Centre, Ido Ekiti, Nigeria.

OLM: Professor of Orthopedics & Chairman Postgraduate Medical Training Committee, Obafemi Awolowo University Teaching Hospitals Complex, Ile- Ife; Nigeria.

OJB: Consultant Orthopedic Surgeon, Federal Medical Centre, Ido Ekiti, Nigeria.
